# Associations between Perceptions and Measures of Weather and Walking, United States—2015

**DOI:** 10.3390/ijerph18168398

**Published:** 2021-08-09

**Authors:** Susan A. Carlson, Geoffrey P. Whitfield, Ryan T. Davis, Erin L. Peterson, Janet E. Fulton, David Berrigan

**Affiliations:** 1 National Center for Chronic Disease Prevention and Health Promotion, Centers for Disease Control and Prevention, Atlanta, GA 30341, USA; clo3@cdc.gov (S.A.C.); xdh5@cdc.gov (G.P.W.); eleepeterson@gmail.com (E.L.P.); jkf2@cdc.gov (J.E.F.); 2 Geospatial Research, Analysis, and Services Program, Division of Toxicology and Human Health Sciences, Agency for Toxic Substances and Disease Registry, Atlanta, GA 30341, USA; ekv5@cdc.gov; 3 Division of Cancer Control and Population Sciences, National Cancer Institute, Bethesda, MD 20892, USA

**Keywords:** walking, physical activity, weather, temperature, humidity, perceptions, perceived barriers, leisure, transportation

## Abstract

Introduction: Weather can be a barrier to walking. Understanding how perceptions of weather as a barrier and measured temperature are associated with walking can inform monitoring and promotion strategies. The objective of this study is to examine the association between perceptions of weather as a barrier to walking and measured weather with the volume of leisure and transportation walking. Methods: The 2015 National Health Interview Survey (NHIS) assessed participation in and volume of walking (transportation, leisure) in the past week and frequency of reporting weather as a barrier to walking. Data were collected over the entire year. In 2019, we merged month-specific temperature data from the PRISM climate group with individual NHIS records. We examined associations using logistic (participation) and linear regression models (volume). Results: Participation in walking increased as frequency of reporting weather as a barrier to walking decreased, from ‘almost always’ (transportation: 23%, leisure: 42%) to ‘a little of the time’ (transportation: 40%, leisure: 67%). Among adults reporting walking, walking volume increased as frequency of reporting weather as a barrier decreased from ‘almost always’ (transportation: 51 min/week, leisure: 64 min/week) to ‘never’ (transportation: 69 min/week, leisure: 98 min/week). Month-specific temperature was significantly associated with leisure walking with lower participation at the lowest and highest temperature quintiles, although the strength of the association differed by frequency of reporting weather as a barrier. Conclusions: In general, prevalence and volume of leisure and transportation walking decreased as the perception of weather as a barrier increased. Low and high temperature conditions were also associated with leisure walking participation, particularly among adults with increased perceptions of weather as a barrier. Our findings highlight the importance of including strategies to help adults overcome perceived and actual weather-related barriers in walking promotion efforts.

## 1. Introduction

Regular physical activity is associated with a reduction in risk of early death and chronic diseases such as coronary heart disease, stroke, type 2 diabetes, depression, and several forms of cancer [1]. Walking is the most commonly reported physical activity among U.S. adults overall, as well as among adults who meet the aerobic physical activity guideline [2,3,4]. Weather, however, can be a barrier to walking [5,6,7]. While weather is not modifiable, there may be strategies to address perceptions of weather as a barrier to walking. Past research on weather, seasonal variation in physical activity and pedestrian behavior has largely focused on measured weather characteristics such as temperature and humidity [8,9,10]. Generally, weather is reported as a barrier to walking and other forms of physical activity with weaker associations between weather and behavior observed for people with greater levels of physical activity and for transportation rather than leisure walking. Few studies have contrasted associations between actual weather and perceptions of weather as a barrier to activity. For example, a qualitative study in older adults from Hong Kong noted respondents view weather as both a barrier and facilitator of walking [11]. A modest amount of research also examines perceptions of temperature and cooling strategies from a more physiological perspective [12,13]. Especially, good and bad weather were associated with leisure time physical activity in a study in Stockholm, Sweden based on travel diaries [14] and travel surveys are beginning to explore weather as an influence on mode choice [15]. In the United States, there have been no prior analyses of national estimates related to perceptions of weather as a barrier to walking and how this differs by demographic and geographic characteristics.

Adults may consider multiple factors, such as actual weather conditions (e.g., temperature, humidity, precipitation), available clothing or indoor alternatives, and their own tolerance to different weather conditions [5,6,7,16,17,18] when determining if weather is a barrier to walking. Measured weather variables for a given area, such as average temperature, can be relatively easy to obtain. Measured weather variables alone, however, may capture only one aspect of this barrier and may not provide a thorough understanding of weather’s influence on transportation and leisure walking. Weather is also widely reported as a barrier to activity [19]. Furthermore, weather is a known risk factor for negative health outcomes. For example, high and low temperatures are associated with cardiopulmonary and other outcomes [20,21,22] and there are winter spikes in mortality in temperate cities [23,24]. It is not clear how people take this information into account when making choices about outdoor activity and it could be used differently for leisure versus transportation or occupation related activity decisions.

The 2015 National Health Interview Survey (NHIS) Cancer Control Supplement collected information about adults’ perceptions of weather as a barrier to walking. These data allowed us to examine the frequency of U.S. adults reporting weather as barrier to walking by select demographic and geographic characteristics. By combining NHIS data with weather data from the PRISM climate group [25], we could examine temperature along with reports of weather as a barrier. This allowed us to examine how perceptions of weather and temperature are associated with transportation and leisure walking and how they may potentially interact. Understanding how these measures together relate to walking can assist in developing methods to monitor this barrier and inform strategies to address weather as a barrier to being physically active.

## 2. Materials and Methods

### 2.1. Survey Description

The NHIS is a multi-stage probability sample survey of U.S. households conducted annually throughout the year and designed to be representative of the civilian, non-institutionalized U.S. population. The NHIS collects basic health and demographic information from all family members. One randomly selected adult (≥18 years of age) answers more questions, including questions from the Cancer Control Supplement about walking behaviors and weather as a barrier. In 2015, the sample adult response rate was 55.2% [26] and as in past years, the distribution of respondents across months is quite consistent (Month, n, %: Jan. 2527, 8.3; Feb. 2496, 8.2; Mar. 2692, 8.8; Apr. 2817, 9.2; May 2752, 9; Jun. 2792, 9.2; Jul. 2712, 8.9; Aug. 2342, 7.7; Sep. 2404, 7.9; Oct. 2379, 7.8; Nov. 2335, 7.7; Dec. 2239, 7.3). Further details are available elsewhere [27].

### 2.2. Measures

Perceptions of weather as a barrier to walking—Respondents were asked “How often does the weather make you less likely to walk? Would you say…” Response options included; almost always, most of the time, some of the time, a little of the time, and never.

Month-specific temperature—Weather variables were created using 2015 data from the PRISM climate group [25]. This group creates time series of climatic datasets for the conterminous United States. We downloaded and analyzed PRISM’s GIS datasets to create a file with a county identifier, month, and mean temperature. At the Research Data Center [28] in 2019, we merged these records with individual NHIS records using the county identifier (a restricted use variable) and month of interview. A categorical variable for average month-specific temperature for each adult was created based on weighted quintiles.

Survey participants are sampled from all 50 U.S States and the District of Columbia. The United States has a largely temperate climate with dry desert environments in the Southwest, a subarctic climate in large parts of Alaska and humid sub-tropical and tropical climates in parts of the southeast, Florida, and Hawaii. Weather in the United States varies widely, with many areas experiencing cold snowy winters and hot summers.

Walking—To assess transportation walking, respondents were asked if they walked ‘to get some place’ for at least 10 min in the past 7 days. To assess leisure walking, respondents were asked if during the past 7 days they walked for at least 10 min ‘for fun, relaxation, exercise, or to walk the dog’. For both transportation and leisure walking, respondents who answered ‘yes’ were asked additional questions about the number of times in the past 7 days they walked (frequency) and the average amount of time they spent walking during an instance (duration). Time spent walking, only among adults reporting walking, was estimated by multiplying frequency by duration.

Demographic characteristics—Age was categorized into five groups (18–24, 25–34, 35–44, 45–64, and ≥65 years). The highest reported grade or year of school completed was categorized into four levels of educational attainment (less than high school, high school graduate, some college, and college graduate). Respondents identified whether they considered themselves Hispanic or Latino, then selected from a list what race or races they consider themselves. Persons who chose more than one race were asked about the one race that ‘best’ described them. Adults were classified into four race/ethnic groups (white, non-Hispanic; black, non-Hispanic; Hispanic; and other, non-Hispanic).

Geographic characteristics—Each respondent’s residence was designated as urban or rural based on 2010 Census urban–rural designation and provided on the dataset as a restricted variable, as previously published [29]. Briefly, urban areas were identified as Census tracts with at least 1000 people/mile^2^ and adjacent tracts with at least 500 people/mile^2^. In addition, a select number of non-residential urban land uses and non-continuous urban developments were identified as urban. Any areas not identified as urban were designated as rural. State of residence was used to categorize individuals into one of nine expanded regions: New England, Middle Atlantic, East North Central, West North Central, South Atlantic, East South Central, West South Central, Mountain, and Pacific. Expanded regions were based on the nine Census divisions [30]; however, similar to other analyses, Delaware, the District of Columbia, and Maryland were moved into the Middle Atlantic division from the South Atlantic [31,32].

### 2.3. Statistical Analysis

Prevalence of reporting frequency of weather as a barrier to walking with corresponding 95% confidence interval were estimated overall, by demographic and geographic characteristics, and by month-specific temperature quintile. Mean month-specific temperature with corresponding 95% confidence interval were estimated overall and by demographic and geographic characteristics. Associations between frequency of reporting weather as a barrier and mean-month-specific temperature with different characteristics were tested with adjusted Wald tests.

For each walking purpose (leisure, transportation), walking was examined using two measures: prevalence (proportion reporting walking for at least 10 min at a time during the past 7 days) and volume (time spent walking among adults reporting walking). Multiple logistic regression models (unadjusted and adjusted) were used to examine the association of month-specific temperature and frequency of reporting weather as a barrier with walking prevalence. The distribution of walking volume among walkers was approximately log-normal. Multiple linear regressions on log-transformed weekly walking volume (unadjusted and adjusted) were used to examine the association of month-specific temperature and frequency of reporting weather as a barrier with walking volume among walkers. To estimate adjusted volume ratios, minutes were back transformed. For logistic and linear regression models, trends across categories of reported weather as a barrier were tested with orthogonal polynomial contrasts. To test for interactions between frequency of reporting weather as a barrier and month-specific temperature, an interaction term was added to the adjusted model. If the interaction term was significant, we presented models stratified by frequency of reporting weather as a barrier to walking. All models adjusted for sex, age group, race/ethnicity, education level, expanded region, and urban–rural designation.

As a sensitivity analysis, we explored additional measures of month-specific weather, including quintiles based on month-specific precipitation and average month-specific Humidex values, a summary index incorporating temperature and humidity. [33] Humidex is calculated using temperature and Dew point and categorized into a comfort scale [34]. It has been widely used in temperate areas of the world including Canada (where it was developed), Italy, and the US [35,36,37]. While Canada is more northerly than the US, summer conditions in Canada span a wide range of temperature and Dew point levels, supporting the use of Humidex in the US. Humidex has also been used in Guangdong, a subtropical region of China [38]. Humidex values have also been linked to occupational injuries, adding to its face validity as a measure of combined thermal and humidity related stress [39] Findings were similar (Supplementary Tables S1–S3) and therefore our main analyses focus only on the simpler and more intuitive measure of temperature.

A total of 33,672 adults were in the publicly-available cancer control supplement. First, 894 adults (2.7%) who reported being unable to walk when responding to the walking question were excluded. Of the remaining 32,778 adults, 7.0% were excluded for missing survey data (0.4% demographic characteristics, 6.2% walking participation or volume, and 0.4% frequency of reporting weather as a barrier) and then 2.5% were excluded for missing month-specific temperature (i.e., weather values were not available in our dataset for adults residing in Alaska or Hawaii). Analyses were performed in Stata 13 (Stata Corporation, College Station, TX, USA) using survey commands to account for the complex survey design and weighting. Statistical tests were deemed significant at *p* < 0.05.

## 3. Results

Frequency of perceiving weather as a barrier to walking significantly differed by sex, age group, education level, region, urban–rural designation, and month-specific temperature quintile (Table 1). The mean month-specific temperatures experienced by U.S. adults were significantly different by race/ethnicity, education level, region, and urban/rural residence. Regional differences were seen with people in the Northeast and Middle Atlantic and East-South Central areas much more likely to report weather as a barrier to walking than those in the Pacific and Mountain areas.

### 3.1. Transportation Walking

Reporting weather as a barrier to walking was significantly associated with transportation walking, both in participation (*p* < 0.001) and volume (*p* < 0.001) (Table 2). Participation in transportation walking increased for those reporting weather as a barrier almost always (23%) to ‘a little of the time’ (40%) (*p*_trend_ < 0.001), while 29% of adults reporting weather was ‘never’ a barrier reported transportation walking. Among those who reported transportation walking, the mean minutes of walking increased as frequency of reporting weather as a barrier decreased (*p*_trend_ < 0.001). The association between month-specific temperature and transportation walking was not significant (participation *p* = 0.084; volume *p* = 0.579). Significant associations between reporting weather as a barrier and transportation walking prevalence and volume remained when models adjusted for demographic and geographic characteristics, and temperature.

### 3.2. Leisure Walking

Reporting weather as a barrier to walking was significantly associated with participation (*p* < 0.001) and volume (*p* < 0.001) of leisure walking (Table 3). Participation in leisure walking increased for those reporting weather as a barrier ‘almost always’ (42%) to ‘a little of the time’ (67%) (*p*_trend_ < 0.001), while 40% of adults reporting weather was ‘never’ a barrier reported leisure walking. Among those who reported leisure walking, the mean minutes of walking increased as frequency of reporting weather as a barrier decreased (*p*_trend_ < 0.001). Significant associations remained when models adjusted for demographic characteristics.

The association between month-specific temperature and leisure walking was significant for participation (*p* < 0.001) and volume (among participants, *p* = 0.017). The prevalence of leisure walking was lowest for the lowest temperature quintile (46%), followed by the highest quintile (50%), and the three middle quintiles had similar prevalence estimates. Among those who reported leisure walking, the mean minutes of walking was similar for those in the lowest two temperature quintiles and the highest three quintiles. Significant associations remained when models adjusted for demographic characteristics.

The association between month-specific temperature and leisure walking (participation and volume) significantly differed by frequency of reporting weather as a barrier (*p*-value for interaction term, participation *p* = 0.004; volume *p* = 0.014; Table 4). The association between temperature and leisure walking was stronger for those who reported weather as a barrier more frequently.

### 3.3. Sensitivity Analysis

Month-specific temperature and Humidex categories were highly correlated and results using Humidex were almost identical to using temperature (see Supplementary Tables S1–S3). We also added mean month-specific precipitation to our models and found it was not significant in any of the models (data not shown).

## 4. Discussion

This study addresses cross-sectional associations of perceptions of weather as a barrier to walking and temperature with self-reported transportation and leisure walking. In general, perceptions of weather as a barrier to walking were negatively associated with volume and prevalence of transportation and leisure walking. Low and high temperature conditions were also associated with lower leisure walking participation, particularly for adults who more frequently perceived weather as a barrier to walking. Our findings highlight the importance of accounting for perceptions of weather as a barrier and the existence of colder and hotter temperatures when developing effective strategies to promote walking.

Our study extends existing epidemiological literature on associations between weather and walking or other physical activity [5,6,17,18,40,41], by examining the two major walking purposes and by including perceived and measured aspects of weather. A recent review of reviews found inconclusive evidence for weather as a correlate of physical activity behavior [41]; however, this review did not present detailed analysis of specific types of physical activity besides walking that could be sensitive to weather and perceptions of weather as barriers. Previous studies support our finding that measured weather may be less influential on transportation or utilitarian walking [6,40]. Less is known about the association between perceptions of weather and walking and whether this association differs by walking purpose. One study examined perceptions of weather using a summative index constructed from responses to the likelihood of four separate items (rainy, cold, hot, and windy weather) inhibiting a respondents walking and found this index to be associated with exercise walking and neighborhood walking, although not significant when examining walking from place to place [9]. Our perceptions question was framed around weather making an individual less likely to walk. The association we observed between our broad question and walking supported its ‘face validity’. Our assessment, however, did not allow us to examine the specific factors contributing to this perception and these may differ by walking types. An increased understanding of what factors influence adults’ perception of weather as a barrier could help develop strategies to address this barrier.

Participation in leisure and transportation walking increased for those reporting weather as a barrier ‘almost always’ to ‘a little of the time’. Surprisingly, this trend did not continue for people reporting weather was ‘never’ a barrier. There may be several reasons why adults selected ‘never’. First, the respondent may not perceive weather as a barrier to walking. However, we would then expect the trend of increasing prevalence of walking with decreasing frequency of reporting weather as a barrier to continue from ‘a little of the time’ to ‘never’; although, this was not the case. A second reason for selecting ‘never’ may be these adults do not walk (for reasons other than weather) and therefore weather is not a perceived barrier. We would expect this issue to dilute the expected association, which is what our data demonstrated. It is likely those selecting the ‘never’ response represent a combination of the two reasons. In future surveys, it may be valuable to offer two response options to help further distinguish between these reasons.

The association between month-specific temperature, perceiving weather as a barrier, and leisure walking was complex. Both measures of weather were significantly associated with leisure walking; however, temperature’s association with leisure walking was dependent on the perception of weather as a barrier. For example, among adults who reported weather as ‘almost always’ a barrier to walking, there was a stronger association between temperature and leisure walking compared to adults who reported weather as a barrier ‘a little of the time’ or ‘never’. A possible explanation is adults who ‘almost always’ perceive weather as a barrier may be more sensitive to the weather and therefore measured temperature has a larger effect on their behavior than those adults reporting less frequently perceiving weather as a barrier. Our findings support the idea that the measure of adult’s perception of weather as a barrier to walking may include multiple factors that are difficult to disentangle. While this presents measurement challenges, it is encouraging from a promotion standpoint as our findings suggest strategies that focus on overcoming weather-related barriers may help promote leisure and transportation walking.

Many strategies can be implemented in communities to promote walking and help adults overcome barriers, including weather. Creation of, or enhancing access to, places for physical activity is a recommended strategy to increase physical activity [42]. Access to indoor facilities, like malls, may provide alternatives for inclement weather [43]. Built environment approaches that combine transportation system interventions with land use and environmental design can make it easier for adults to get from place to place [44]. By making walking easier overall, concerns about weather may be less influential. In addition, incorporating specific features—such as shade trees, building awnings, and covered public transit shelters—may help to overcome weather challenges and providing social support [42] may provide the encouragement needed to help individuals overcome barriers. Individuals can also be encouraged to wear appropriate clothing, walk during times that minimize weather extremes, or pursue a different physical activity when outdoor walking is challenging [7]. Specific groups may be more susceptible to weather barriers and the role demographics play is important to consider when developing promotion strategies [16]. For example, strategies to address weather as a barrier to walking may be particularly important for women and older adults who have a higher prevalence of ‘almost always’ perceiving weather as a barrier to walking.

This study is subject to several limitations. First, the analysis is based on self-report of walking and may be biased [45]; however, we do not think this bias would influence the association between weather and walking. Second, information about walking for bouts less than 10 min was not collected. This could lead to underestimates of total walking. Weather may have less influence on short bouts so our findings may have changed if we captured shorter bouts. Third, there is a mismatch between our perceptions of weather as a barrier and month-specific temperature. The perception question does not prompt the responder to consider a specific time-period. Fourth, we do not know if the walking reported was inside or outside. While most transportation walking would most likely be outside, there are options for indoor leisure walking such as shopping malls or indoor tracks. Associations between weather and walking may differ for indoor and outdoor walking; however, our data did not allow us to examine this. Fifth, the survey response rate was 55.2% and about 6% of respondents dropped out prior to completing the NHIS Cancer Control Supplement. This could contribute to response bias if non-responders differed systematically from responders. However, survey weights may help to reduce some of the influence of non-response.

Exploring regional differences in reporting weather as a barrier to walking is an important area for future research. In this study we included expanded census regions as a covariate to broadly account for different social and climatic characteristics of the United States. These regions differ somewhat from the nine US climate regions identified by the NOAA National Centers for Environmental Information (https://www.ncdc.noaa.gov/monitoring-references/maps/us-climate-regions.php; accessed on 8 June 2021). Our focus in this paper is on weather and walking during specific months. Future studies could examine whether broader climate regions might interact with specific weather characteristics to influence perception of weather as a barrier to walking. We further note that the climate regions of the US include very different kinds of weather, with arid deserts of Nevada in the same region as heavily forested Northern California and temperate Virginia in the same region as sub-tropical and tropical Florida. The National Health Interview features a single question about weather as a barrier to walking. Future studies could further explore differential influences of heat and cold on the perception of weather as a barrier to walking since race, age, and education appear to interact with weather to influence choices between indoor and outdoor exercise [18]. For example, the highest reports of weather as a barrier to walking in this study were from the Northeast and Middle Atlantic, notably cold and windy in the winter and the East South Central, an area that is very hot and humid during a large portion of the year. However, weather alone may not be the only explanation for regional differences in the perception of weather as a barrier since there are many underlying social and attitudinal differences between these regions and analysis of American Time Use Survey data in relation to climate regions suggest that both culture and weather influence walking in the US [46].

A second topic for future analyses involves associations between air pollution and walking. Elevated levels of air pollution may reduce physical activity and walking even though most studies have suggested that the benefits of physical activity outweigh the risks of additional pollution exposure associated with activity [47]. One study in Korea has shown that precipitation, but not low temperatures, are associated with pedestrian volume in models including PM10 concentrations [48]. Better understanding risk perceptions and comfort might help further explain the joint effects of weather and air pollution on walking.

This study has several strengths. First, it is based on a nationally representative sample. The large sample size and the richness of the data collected enabled multiple and stratified analysis. Second, we had information on leisure and transportation walking and the perception of weather as a barrier to walking. Third, by doing the analysis at the Research Data Center, we could merge in temperature data at the county level, and include additional geographic identifiers in our model, including expanded region and urban–rural designation.

## 5. Conclusions

Regardless of walking purpose, prevalence, and volume of walking generally increased as perceptions of weather as a barrier decreased in frequency. Low and high temperature conditions were also associated with lower leisure walking participation, particularly among adults with increased perceptions of weather as a barrier. Our findings highlight the importance of accounting for perceptions of weather as a barrier and the existence of colder and hotter temperatures conditions when developing effective strategies to promote walking.

## Figures and Tables

**Table 1:** Frequency of reporting weather as a barrier to walking and mean temperature by select characteristics.

Characteristics	Frequency of Reporting Weather as a Barrier to Walking (%, 95% CI)										Mean Temperature (°C) (Mean, 95% CI)	
	Almost Always		Most of the Time		Some of the Time		a Little of the Time		Never			
Total	20.8	20.0–21.7	13.3	12.7–13.8	26.4	25.6–27.2	15.7	15.0–16.3	23.8	23.0–24.6	14.4	14.2–14.7
**Sex**												
Men	18.0	17.0–19.1	12.5	11.7–13.3	24.8	23.8–25.9	16.9	15.9–17.8	27.8	26.7–29.0	14.3	14.0–14.7
Women	23.5	22.4–24.7	14.0	13.3–14.8	27.9	26.9–28.9	14.5	13.7–15.3	20.1	19.1–21.0	14.5	14.2–14.8
**Age group (years)**												
18–24	16.6	14.7–18.7	15.1	13.2–17.2	26.7	24.3–29.1	16.9	14.9–19.1	24.7	22.5–27.0	14.6	14.0–15.2
25–34	19.1	17.6–20.7	13.5	12.4–14.8	29.6	28.0–31.3	16.3	15.0–17.7	21.4	19.9–22.9	14.5	14.1–14.9
35–44	19.9	18.3–21.6	13.6	12.4–14.9	27.0	25.3–28.7	16.1	14.7–17.6	23.4	21.8–25.0	14.4	13.9–14.8
45–64	20.6	19.4–21.9	13.1	12.2–14.1	26.6	25.4–27.8	15.7	14.7–16.8	24.0	22.8–25.4	14.3	13.9–14.6
≥65	26.7	25.1–28.3	11.8	10.8–12.9	22.3	21.0–23.6	13.6	12.5–14.8	25.6	24.1–27.1	14.6	14.2–15.0
**Race/ethnicity**												
White, non-Hispanic	21.4	20.4–22.5	13.4	12.7–14.1	26.5	25.5–27.5	15.8	15.1–16.7	22.8	21.8–23.8	13.5	13.2–13.9
Black, non-Hispanic	22.9	20.9–25.0	13.2	11.9–14.6	24.5	22.7–26.4	14.0	12.5–15.7	25.4	23.4–27.5	15.7	15.2–16.2
Hispanic	18.7	17.1–20.4	12.5	11.3–13.8	24.8	23.2–26.5	16.2	14.8–17.6	27.9	26.1–29.7	17.0	16.6–17.5
Other, non-Hispanic	17.2	15.2–19.3	13.9	12.1–16.1	31.8	29.3–34.4	15.3	13.4–17.4	21.8	19.7–24.1	14.8	14.3–15.4
**Education level**												
Less than high school	22.5	20.7–24.5	12.6	11.3–14.1	22.9	21.2–24.7	14.1	12.5–15.9	27.9	25.9–29.8	15.2	14.7–15.7
High school graduate ^a^	23.1	21.6–24.6	13.5	12.4–14.7	24.4	23.0–25.8	12.8	11.8–13.9	26.2	24.8–27.7	14.6	14.2–15.0
Some college	21.2	20.0–22.6	13.3	12.3–14.3	26.5	25.1–27.9	15.2	14.2–16.3	23.8	22.5–25.1	14.3	14.0–14.7
College graduate	18.1	16.9–19.4	13.4	12.5–14.4	29.2	28.0–30.5	18.8	17.7–20.0	20.5	19.3–21.7	14.1	13.7–14.5
**Expanded region ^b^**												
New England	26.2	22.5–30.2	11.0	9.1–13.3	27.4	24.0–31.0	14.2	11.3–17.6	21.3	18.4–24.5	9.1	7.6–10.7
Middle Atlantic	25.5	23.3–27.9	15.9	14.3–17.8	27.2	25.2–29.3	12.2	10.8–13.8	19.1	17.2–21.1	11.8	11.0–12.5
East North Central	21.9	19.8–24.1	15.5	14.1–17.1	30.2	28.0–32.5	13.6	12.3–14.9	18.8	17.1–20.6	10.2	9.4–11.1
West North Central	21.5	19.0–24.3	18.2	16.4–20.1	29.4	26.2–32.8	13.3	11.3–15.5	17.6	15.2–20.3	10.6	9.4–11.8
South Atlantic	21.8	19.4–24.4	11.9	10.7–13.1	24.4	22.8–26.2	16.5	14.9–18.3	25.3	23.3–27.5	19.0	18.5–19.6
East South Central	27.9	24.0–32.2	13.5	11.2–16.1	23.0	20.2–26.0	11.9	10.0–13.9	23.8	20.7–27.2	15.4	14.4–16.5
West South Central	21.8	18.6–25.5	12.3	10.8–14.1	24.4	22.3–26.6	17.6	15.4–20.0	23.8	21.1–26.8	18.9	18.2–19.6
Mountain	15.4	13.4–17.8	12.1	10.4–14.0	32.0	29.6–34.6	18.3	16.0–20.9	22.1	19.8–24.7	12.9	12.0–13.8
Pacific	11.7	10.4–13.2	9.7	8.6–11.0	22.9	21.2–24.6	20.2	18.5–22.1	35.5	33.4–37.6	16.2	15.8–16.6
**Urban–rural designation**												
Urban	20.2	19.3–21.1	13.3	12.7–13.9	26.6	25.8–27.4	16.1	15.4–16.8	23.9	23.1–24.7	14.7	14.4–15.0
Rural	23.6	21.6–25.8	13.4	12.1–14.8	25.7	23.7–27.8	13.7	12.1–15.5	23.6	21.5–25.7	13.4	12.7–14.1
**Temperature quintile**												
<6.7 °C	26.1	24.3–28.0	17.1	15.7–18.5	27.4	25.8–29.1	11.2	10.1–12.4	18.2	16.8–19.7	−0.4	−0.8–0.0
6.7 to <12.6 °C	20.4	18.7–22.3	14.1	12.8–15.6	27.6	25.8–29.3	16.3	15.0–17.7	21.6	20.0–23.2	9.9	9.8–10.0
12.6 to <18.9 °C	17.7	16.2–19.3	11.1	10.0–12.4	25.3	23.6–27.0	18.9	17.3–20.7	27.0	25.1–28.9	15.8	15.7–16.0
18.9 to <22.9 °C	17.2	15.6–19.0	12.2	11.1–13.5	27.5	25.8–29.3	16.6	15.3–18.0	26.4	24.6–28.2	21.0	20.9–21.1
≥22.9 °C	22.8	20.8–24.9	11.8	10.7–13.0	24.2	22.6–25.9	15.2	13.7–16.7	26.0	24.1–28.0	26.0	25.9–26.1

Boldface indicates statistical significance (*p* < 0.05). ^a^ Includes individuals with a General Educational Development test or equivalent. ^b^ Expanded region was categorized as New England (Connecticut, Maine, Massachusetts, New Hampshire, Rhode Island, and Vermont), Middle Atlantic (Delaware, Washington DC, Maryland, New Jersey, New York, and Pennsylvania), East North Central (Illinois, Indiana, Michigan, Ohio, and Wisconsin); West North Central (Iowa, Kansas, Minnesota, Missouri, Nebraska, North Dakota, and South Dakota), South Atlantic (Florida, Georgia, North Carolina, South Carolina, Virginia, and West Virginia); East South Central (Alabama, Kentucky, Mississippi, and Tennessee); West South Central (Arkansas, Louisiana, Oklahoma, and Texas); Mountain (Arizona, Colorado, Idaho, Montana, Nevada, New Mexico, Utah, and Wyoming); and Pacific (California, Oregon, and Washington). Alaska and Hawaii are not listed in the Pacific region as residence from these states were excluded because weather data were not available.

**Table 2:** Prevalence and volume (among participants) of transportation walking by weather measure.

Weather Measure	Participation						Volume among Participants					
	%	95% CI	Model 1 ^a^		Model 2 ^b^		Minutes/Week		Model 1 ^a^		Model 2 ^b^	
			PR	95% CI	PR	95% CI	Mean	95% CI	VR	95% CI	VR	95% CI
Total	**31.5**	30.7–32.4					59.3	57.6–61.0				
**Frequency of reporting weather as a barrier**												
Almost always	23.2	21.7–24.7	1.00	Referent	1.00	Referent	51.2	47.5–54.8	1.00	Referent	1.00	Referent
Most of the time	32.0	29.8–34.1	**1.30**	**1.19–1.42**	**1.30**	**1.18–1.41**	50.5	47.1–54.0	1.00	0.91–1.10	1.00	0.91–1.10
Some of the time	35.2	33.7–36.8	**1.42**	**1.31–1.52**	**1.41**	**1.31–1.52**	58.4	55.4–61.3	**1.15**	**1.04–1.25**	**1.14**	**1.04–1.25**
A little of the time	40.2	38.1–42.3	**1.58**	**1.46–1.71**	**1.57**	**1.45–1.70**	64.6	60.3–68.9	**1.29**	**1.16–1.41**	**1.28**	**1.16–1.40**
Never	28.8	27.2–30.4	**1.16**	**1.07–1.25**	**1.15**	**1.06–1.24**	68.6	64.3–73.0	**1.35**	**1.22–1.47**	**1.34**	**1.22–1.47**
**Temperature quintile**												
<6.7 °C	30.1	28.2–32.0	1.00	Referent	1.00	Referent	56.7	53.0–60.5	1.00	Referent	1.00	Referent
6.7 to <12.6 °C	34.3	32.2–36.5	**1.13**	**1.03–1.22**	**1.10**	**1.01–1.20**	60.7	56.8–64.6	1.07	0.97–1.17	1.06	0.96–1.16
12.6 to <18.9 °C	31.9	30.0–33.9	1.06	0.97–1.15	1.04	0.95–1.12	60.3	56.5–64.0	1.06	0.96–1.16	1.04	0.94–1.13
18.9 to <22.9 °C	32.2	30.1–34.3	1.08	0.99–1.17	1.05	0.97–1.14	60.1	56.0–64.2	1.07	0.96–1.17	1.04	0.94–1.14
≥22.9 °C	29.0	27.2–30.9	1.04	0.95–1.14	1.04	0.95–1.13	58.5	54.3–62.6	1.03	0.92–1.13	1.01	0.90–1.11

Boldface indicates statistical significance (*p* < 0.05). ^a^ Model 1 includes sex, age group, race/ethnicity, education level, expanded region, urban–rural designation, and the weather variable of interest. ^b^ Model 2 includes all demographic and geographic variables in Model 1 and both measures of weather.

**Table 3:** Prevalence and volume of leisure walking (among participants) by weather measure.

Weather Measure	Participation				Volume among Participants			
	%	95% CI	Adjusted ^a^		Minutes/Week		Adjusted ^a^	
			PR	95% CI	Mean	95% CI	VR	95% CI
**Total**	**52.1**	51.2–53.0			79.7	78.1–81.3		
**Frequency of reporting weather as a barrier**								
Almost always	41.8	40.0–43.6	1.00	Referent	63.6	60.5–66.6	1.00	Referent
Most of the time	52.9	50.6–55.2	**1.23**	**1.16–1.30**	66.5	62.9–70.0	1.06	0.99–1.14
Some of the time	61.8	60.2–63.3	**1.41**	**1.35–1.48**	78.3	75.6–81.1	**1.25**	**1.17–1.32**
A little of the time	66.7	64.7–68.7	**1.51**	**1.43–1.59**	92.2	87.9–96.5	**1.47**	**1.38–1.57**
Never	40.4	38.7–42.0	0.94	0.89–0.99	97.9	93.5–102.3	**1.58**	**1.47–1.69**
**Temperature quintile**								
<6.7 °C	45.8	43.9–47.7	1.00	Referent	76.3	72.5–80.1	1.00	Referent
6.7 to <12.6 °C	53.2	51.1–55.3	**1.15**	**1.09–1.21**	76.4	73.0–79.9	1.02	0.95–1.08
12.6 to <18.9 °C	55.4	53.5–57.4	**1.18**	**1.12–1.25**	82.7	79.1–86.3	**1.09**	**1.02–1.17**
18.9 to <22.9 °C	55.7	53.9–57.5	**1.21**	**1.15–1.27**	81.3	77.8–84.7	**1.08**	**1.01–1.15**
≥22.9 °C	50.3	48.3–52.4	**1.13**	**1.07–1.20**	81.3	77.4–85.3	**1.10**	**1.02–1.18**

Boldface indicates statistical significance (*p* < 0.05). ^a^ Model includes sex, age group, race/ethnicity, education level, expanded region, urban-rural designation, and the weather variable of interest.

**Table 4:** Prevalence and volume of leisure walking (among participants) by frequency of reporting weather as a barrier by temperature quintile.

Frequency by Temperature Quintile	Participation				Volume among Participants			
	%	95% CI	Adjusted ^a^		Minutes/Week		Adjusted ^a^	
			PR	95% CI	Mean	95% CI	VR	95% CI
**Almost always**								
<6.7 °C	31.8	28.6–35.0	1.00	Referent	54.0	47.6–60.5	1.00	Referent
6.7 to <12.6 °C	44.3	40.3–48.2	**1.42**	**1.22–1.61**	59.1	53.1–65.2	1.12	0.94–1.29
12.6 to <18.9 °C	48.5	44.2–52.8	**1.55**	**1.34–1.75**	69.1	61.0–77.2	**1.35**	**1.13–1.57**
18.9 to <22.9 °C	50.3	45.8–54.8	**1.62**	**1.41–1.83**	67.8	61.2–74.3	**1.30**	**1.10–1.50**
≥22.9 °C	39.2	35.1–43.2	**1.29**	**1.10–1.49**	69.1	61.8–76.4	**1.34**	**1.11–1.57**
**Most of the time**								
<6.7 °C	46.1	41.8–50.5	1.00	Referent	61.0	53.3–68.7	1.00	Referent
6.7 to <12.6 °C	55.4	50.5–60.3	**1.17**	**1.02–1.31**	64.8	57.2–72.4	1.07	0.89–1.24
12.6 to <18.9 °C	56.7	51.6–61.7	**1.21**	**1.06–1.36**	72.3	63.8–80.7	1.16	0.96–1.35
18.9 to <22.9 °C	57.8	52.6–62.9	**1.23**	**1.08–1.38**	70.3	62.6–78.0	1.14	0.95–1.33
≥22.9 °C	50.9	45.9–55.9	1.09	0.93–1.26	65.8	57.6–74.1	1.04	0.85–1.24
**Some of the time**								
<6.7 °C	56.1	52.8–59.3	1.00	Referent	77.6	72.2–83.0	1.00	Referent
6.7 to <12.6 °C	61.6	58.1–65.2	1.09	0.999–1.17	79.6	73.4–85.8	1.03	0.92–1.13
12.6 to <18.9 °C	63.4	60.0–66.8	**1.11**	**1.02–1.20**	78.4	72.3–84.5	1.01	0.90–1.13
18.9 to <22.9 °C	65.9	62.6–69.2	**1.16**	**1.07–1.25**	80.9	74.8–87.0	1.04	0.93–1.15
≥22.9 °C	61.9	58.4–65.5	**1.12**	**1.03–1.22**	74.5	67.9–81.0	0.97	0.86–1.08
**A little of the time**								
<6.7 °C	63.5	58.6–68.4	1.00	Referent	96.4	86.2–106.7	1.00	Referent
6.7 to <12.6 °C	68.2	64.0–72.4	1.10	0.99–1.21	90.5	81.5–99.5	0.99	0.85–1.13
12.6 to <18.9 °C	67.9	63.9–71.8	1.08	0.97–1.19	95.0	86.1–103.8	1.01	0.87–1.16
18.9 to <22.9 °C	66.9	62.7–71.1	1.08	0.97–1.19	85.0	76.2–93.7	0.89	0.76–1.02
≥22.9 °C	66.1	61.2–70.9	**1.13**	**1.01–1.24**	96.3	85.1–107.4	1.05	0.88–1.23
**Never**								
<6.7 °C	39.0	34.9–43.2	1.00	Referent	111.5	97.0–125.9	1.00	Referent
6.7 to <12.6 °C	38.2	34.3–42.1	0.91	0.78–1.03	86.5	77.3–95.7	0.82	0.69–0.96
12.6 to <18.9 °C	43.3	39.8–46.8	0.95	0.82–1.08	93.6	85.5–101.6	0.91	0.76–1.06
18.9 to <22.9 °C	40.6	37.2–44.0	0.98	0.86–1.10	99.6	90.2–109.1	0.96	0.81–1.11
≥22.9 °C	39.9	36.2–43.5	0.97	0.84–1.10	102.1	92.1–112.2	1.01	0.84–1.18

PR, Prevalence ratio; VR, Volume ratio. Boldface indicates statistical significance (*p* < 0.05). ^a^ Models include sex, age group, race/ethnicity, education level, expanded region, urban–rural designation, and temperature, and models are stratified by reported weather as a barrier to walking.

## Data Availability

The data analyzed in this study are publicly available at https://www.cdc.gov/nchs/nhis/index.htm and https://prism.oregonstate.edu, accessed on 7 August 2021.

## Supplementary Materials

The following are available online at https://www.mdpi.com/article/10.3390/ijerph18168398/s1, Table S1: Prevalence and volume of transportation walking by weather as a barrier and monthly Humidex quintile; Table S2: Prevalence and volume of leisure walking by weather as a barrier to walking and monthly Humidex quintile; Table S3: Prevalence and volume of leisure walking by weather as a barrier and monthly Humidex quintile.
