# Supplementary material for: Associations between Perceptions and Measures of Weather and Walking, United States—2015
Source: Int J Environ Res Public Health. 2021 Aug 9;18(16):8398. doi: 10.3390/ijerph18168398 (PMC8392542; doi:10.3390/ijerph18168398)
Supplement: Supplementary file 1 [file ijerph-18-08398-s001.zip › ijerph-1307047-SI.pdf]

Supplemental Tables

Table S1. Prevalence and volume of transportation walking by weather as a barrier and monthly Humidex quintile.

	**Participation**						**Volume Among Adults Transportation Walking**					
	**%**	**95% CI**	**Model 1[a]**		**Model 2[b]**		**min/wk**		**Model 1[a]**		**Model 2[b]**	
**Characteristics**			**PR**	**95% CI**	**PR**	**95% CI**	**Mean**	**95% CI**	**VR**	**95% CI**	**VR**	**95% CI**
Total	31.5	30.7-32.4	--		--		59.3	57.6-61.0	--		--	
Frequency of reporting weather as a barrier												
Almost always	23.2	21.7-24.7	1.00	Referent	1.00	Referent	51.2	47.5-54.8	1.00	Referent	1.00	Referent
Most of the time	32.0	29.8-34.1	**1.30**	**1.19-1.42**	**1.30**	**1.18-1.41**	50.5	47.1-54.0	1.00	0.91-1.1	1.00	0.91-1.1
Some of the time	35.2	33.7-36.8	**1.42**	**1.31-1.52**	**1.41**	**1.31-1.52**	58.4	55.4-61.3	**1.15**	**1.04-1.25**	**1.14**	**1.04-1.25**
A little of the time	40.2	38.1-42.3	**1.58**	**1.46-1.71**	**1.58**	**1.45-1.70**	64.6	60.3-68.9	**1.29**	**1.16-1.41**	**1.28**	**1.16-1.41**
Never	28.8	27.2-30.4	**1.16**	**1.07-1.25**	**1.15**	**1.06-1.24**	68.6	64.3-73.0	**1.35**	**1.22-1.47**	**1.35**	**1.22-1.47**
Humidex quintile[c]												
1	30.0	28.1-31.9	1.00	Referent	1.00	Referent	57.5	53.7-61.2	1.00	Referent	1.00	Referent
2	34.9	32.8-37.1	**1.13**	**1.04-1.23**	**1.11**	**1.01-1.20**	60.4	56.5-64.4	1.05	0.95-1.15	1.04	0.94-1.13
3	32.0	30.1-34.0	1.05	0.96-1.14	1.03	0.94-1.12	60.5	56.9-64.2	1.05	0.95-1.14	1.03	0.93-1.12
4	32.2	30.1-34.4	1.07	0.98-1.16	1.05	0.96-1.14	59.2	55.2-63.3	1.04	0.94-1.14	1.01	0.92-1.11
5	28.4	26.6-30.2	1.05	0.96-1.14	1.04	0.95-1.13	58.6	54.3-62.9	1.03	0.91-1.14	1.01	0.90-1.12

PR, Prevalence ratio; VR, Volume ratio

Boldface indicates statistical significance (p<0.05).

[a]Model 1 includes sex, age group, race/ethnicity, education level, expanded region, urban-rural designation, and the weather variable of interest.
[b]Model 2 includes all demographic and geographic variables in model 1 and both measures of weather.
[c]Upper bounds for Humidex quintiles are: 1: 4.580; 2: 11.911; 3: 20.491; 4: 26.701.

Table S2. Prevalence and volume of leisure walking by weather as a barrier to walking and monthly Humidex quintile.

Characteristics	Participation				Volume Among Adults Leisure Walking			
	%	95% CI	Adjusted[a]		min/wk		Adjusted[a]	
			PR	95% CI	Mean	95% CI	VR	95% CI
Total	31.5	30.7-32.4	--		59.3	57.6-61.0	--	
Frequency of reporting weather as a barrier								
Almost always	41.8	40.0-43.6	1.00	Referent	63.6	60.5-66.6	1.00	Referent
Most of the time	52.9	50.6-55.2	**1.23**	**1.16-1.3**	66.5	62.9-70.0	1.06	0.99-1.14
Some of the time	61.8	60.2-63.3	**1.41**	**1.35-1.48**	78.3	75.6-81.1	**1.25**	**1.17-1.32**
A little of the time	66.7	64.7-68.7	**1.51**	**1.43-1.59**	92.2	87.9-96.5	**1.47**	**1.38-1.57**
Never	40.4	38.7-42.0	0.94	0.89-0.99	97.9	93.5-102.3	**1.58**	**1.47-1.69**
Humidex quintile[b]								
1	45.8	43.9-47.7	1.00	Referent	75.3	71.6-78.9	1.00	Referent
2	53.5	51.4-55.5	**1.15**	**1.09-1.21**	78.7	75.0-82.4	1.05	0.98-1.12
3	55.2	53.2-57.2	**1.17**	**1.11-1.24**	81.2	77.7-84.7	**1.08**	**1.01-1.16**
4	56.5	54.7-58.3	**1.22**	**1.16-1.28**	82.0	78.3-85.7	**1.10**	**1.03-1.18**
5	49.5	47.5-51.6	**1.12**	**1.06-1.19**	80.6	76.8-84.4	**1.11**	**1.03-1.19**

PR, Prevalence ratio; VR, Volume ratio

Boldface indicates statistical significance (p<0.05).

[a]Model includes sex, age group, race/ethnicity, education level, expanded region, urban-rural designation, and the weather variable of interest.

[b]Upper bounds for Humidex quintiles: 1: 4.580; 2: 11.911; 3: 20.491; 4: 26.701.

Table S3. Prevalence and volume of leisure walking by weather as a barrier and monthly Humidex quintile.

Frequency of reporting weather as a barrier by humidex quintile[b]	Participation				Volume Among Adults Leisure Walking			
	%	95%CI	Adjusted[a]		min/week		Adjusted[a]	
			PR	95% CI	Mean	95%CI	VR	95% CI
Almost always								
1	32.0	29.0-35.2	1.00	Referent	53.8	47.4-60.1	1.00	Referent
2	44.9	40.9-49.0	**1.43**	**1.24-1.62**	60.0	53.8-66.2	**1.14**	**0.96-1.31**
3	47.0	42.7-51.3	**1.50**	**1.29-1.71**	66.9	58.8-75.0	**1.31**	**1.10-1.53**
4	48.8	44.3-53.4	**1.57**	**1.36-1.78**	70.6	63.7-77.5	**1.36**	**1.15-1.56**
5	40.9	37.0-44.9	**1.34**	**1.15-1.54**	67.9	61.1-74.8	**1.32**	**1.10-1.54**
Most of the time								
1	46.1	41.8-50.5	1.00	Referent	59.1	51.9-66.2	1.00	Referent
2	55.1	50.2-59.9	**1.15**	**1.01-1.30**	67.5	59.1-75.9	1.14	0.95-1.34
3	58.4	53.2-63.4	**1.26**	**1.10-1.41**	69.5	62.2-76.9	1.15	0.97-1.33
4	56.6	51.4-61.6	1.19	1.04-1.34	70.1	61.6-78.5	1.18	0.98-1.39
5	51.2	46.2-56.1	1.11	0.95-1.27	68.1	59.9-76.4	1.13	0.93-1.34
Some of the time								
1	56.4	53.2-59.6	1.00	Referent	77.7	72.3-83.2	1.00	Referent
2	60.7	57.0-64.3	1.05	0.97-1.14	80.2	74.0-86.3	1.02	0.92-1.13
3	63.5	60.1-66.7	**1.10**	**1.02-1.18**	78.8	72.7-84.8	1.02	0.91-1.14
4	66.6	63.2-69.9	**1.16**	**1.08-1.25**	79.1	72.8-85.4	1.01	0.90-1.12
5	61.7	58.1-65.3	**1.11**	**1.02-1.20**	75.6	69.1-82.1	0.98	0.87-1.10
A little of the time								
1	64.3	59.2-69.0	1.00	Referent	95.7	85.7-105.7	1.00	Referent
2	68.8	64.5-72.8	1.08	0.98-1.19	92.4	82.0-102.7	1.00	0.85-1.15
3	67.4	63.1-71.3	1.05	0.95-1.16	93.5	85.2-101.8	1.00	0.85-1.14
4	68.5	64.1-72.6	1.09	0.98-1.20	88.3	78.6-98.0	0.94	0.80-1.08
5	63.8	58.8-68.4	1.08	0.97-1.19	92.6	82.9-102.3	1.01	0.85-1.17
Never								
1	38.1	34.2-42.2	1.00	Referent	107.4	93.8-121.0	1.00	Referent

2	39.9	36.1-43.8	0.95	0.82-1.07	93.3	83.1-103.5	0.91	0.76-1.06
3	43.3	40.0-46.7	0.95	0.83-1.07	90.8	83.1-98.5	0.91	0.76-1.06
4	43.5	40.1-47.0	1.06	0.93-1.19	98.9	90.1-107.6	1.01	0.85-1.17
5	35.8	32.3-39.5	0.93	0.80-1.07	104.3	92.4-116.2	1.05	0.86-1.24

PR, Prevalence ratio; VR, Volume ratio

Boldface indicates statistical significance ($p<0.05$).

[a]Models include sex, age group, race/ethnicity, education level, expanded region, urban-rural designation, and temperature, and models are stratified by reported weather as a barrier to walking.
[b]Upper bounds for Humidex quintiles: 1: 4.580; 2: 11.911; 3: 20.491; 4: 26.701.
